# Bestrophin 2 is expressed in human non-pigmented ciliary epithelium but not retinal pigment epithelium

**Published:** 2010-02-10

**Authors:** Youwen Zhang, Rajkumar V. Patil, Alan D. Marmorstein

**Affiliations:** 1 Department of Ophthalmology and Vision Science, University of Arizona, Tucson, AZ; 2 Pharmaceutical Research, Alcon Laboratories, Fort Worth, TX; 3 College of Optical Sciences, University of Arizona, Tucson, AZ

## Abstract

**Purpose:**

Mice in which bestrophin 2 (*Best2*) is disrupted exhibit changes in aqueous flow and drainage, resulting in a reduction in intraocular pressure in comparison to wild-type mice. *Best2* encodes a putative anion channel localized uniquely to the basolateral plasma membrane of non-pigmented epithelium cells in mice. In this study, we examine the localization of Best2 in the human eye.

**Methods:**

Rabbit polyclonal antibodies recognizing human Best2 (hBest2) were generated and characterized for use in western blot, immunoprecipitation, and immunofluorescence assays. The expression of hBest2 using these antibodies was examined using human donor eye tissues.

**Results:**

We could not detect hBest2 in human ciliary bodies or other ocular tissues by western blot. However, when enriched by immunoprecipitation, hBest2 was identified in ciliary bodies, but not in the retinal pigment epithelium. Using immunofluorescence, we located hBest2 in the basolateral plasma membrane of non-pigmented epithelial cells.

**Conclusions:**

We found expression of hBest2 similar to mice only in NPE cells. These data suggest that Best2 may play a functional role in the regulation of aqueous flow and drainage in humans. We conclude that Best2 represents a new potential target for glaucoma therapy.

## Introduction

Bestrophin 2 is a member of the Bestrophin/RFP-TM family of proteins [1,2]. There are four paralogous groups of bestrophin genes in mammals, designated *Best1* through *Best4.* In the mouse, *Best4* is a pseudogene [3]. There is little information on *BEST4* in any species [1]. Only Best1 and Best2 are known to be expressed in the eye [3–5]. In all species examined to date, Best1 is localized exclusively in the basolateral plasma membrane of the retinal pigment epithelium (RPE) cells [5–9]. The localization of Best2, however, is known only in the mouse [4]. Using mice in which the *Best2* gene was disrupted by insertion of a *Lac Z* reporter, we found that *Best2* gene expression is strongest in the non-pigmented epithelium (NPE) cells of the eye and in colon epithelia [4]. Antibodies specific to mouse Best2 (mBest2) confirmed these findings and showed that mBest2 is localized to the basolateral plasma membrane of those cells. mBest2 is also expressed in the olfactory epithelium [10,11] and in salivary acinar cells [1].

The function of the bestrophins is poorly understood [1]. While there is evidence that bestrophins function as Cl^-^ channels [12,13], this function is inconsistent with the phenotypes of *Best1* [14] and *Best2* [4,15] knockout mice, or knock-in mice carrying the dominant Best vitelliform macular dystrophy disease-causing mutation Best1^W93C^ [16]. None of these mouse strains have a deficit in whole-cell Cl^-^ conductances in tissues normally expressing the protein, though defects in Ca^2+^ signaling are found in *Best1^−/−^* and *Best1^W93C^* knock-in mice [14,16].

Mutations in *BEST1* are causally associated with five human retinal degenerative diseases [1,17]. Although mutations in *Best2* are not known to cause disease, the *Best2* null mouse was found to have a significantly reduced intraocular pressure (IOP) when compared to heterozygous and wild-type littermates [4]. In a follow-up to that work, we demonstrated that lack of Best2 results in an increase in aqueous flow and in drainage via the conventional and uveoscleral pathways [18]. Recent work in our laboratory and others has linked bestrophins to regulation of voltage-dependent Ca^2+^ channels [14,19–21], and has found a significant permeability of Bestrophin channels to bicarbonate [22]. The latter function could explain the phenotype of the *Best2* null mouse, and explain the apparent synergistic effect on IOP of carbonic anhydrase inhibitors and lack of Best2 [4]. This phenotype suggests that Best2 could be an attractive target for diminishing IOP in individuals with glaucoma. However, little is known about Best2 in humans. As such, our goal in this study was to determine whether hBest2, like mBest2, is exclusively located in NPE cells in the eye.

## Methods

### Plasmid vectors

A full-length coding sequence for *BEST2* in pCMV6XL5 was obtained from Origene (Rockville, MD). The *BEST2* coding sequence was subcloned into SalI and BamHI sites of pAdlox, following PCR, with the primers 5′-ATC AGT CGA CAT GAC CGT CAC CTA CAC AGC C-3′ and 5′-ATC AGG ATC CTC AGG CCA GAT TCT CCT CCT C-3′. pAdlox-hBest1, pEGFP-mBest1, and pCMV-mBest2 have been described elsewhere [5]. pCDNA3.1–mBest3 and pRK5-hBest3 were the kind gift of Dr. H. Criss Harzell (Emory University, Atlanta, GA)

### Production of anti-hBest2

An anti-hBest2 antiserum (GA3512) was produced using the proprietary Genomic Antibody Technology™ process (Strategic Design Inc., Newark, DE) in rabbits, using the following amino acid sequence: PAGAGMVAGG PLGRRLSFLL RKNSCVSEAS TGASCSCAVV PEGAAPECSC GDPLLDPGLP EPEAPPPAGP EPLTLIPGPV EPFSIVTMPG PRGPAPPWLP. This sequence corresponds to amino acids 399–498 of hBest2 (NM_017682).

Other antibodies used in this study included affinity-purified rabbit anti-mBest2 (B4947A) [4], affinity-purified rabbit anti-mBest1 (Pab-003) [5], rabbit anti-mBest3 (05619) antibodies (obtained from Dr. H.C. Hartzell, Emory University, Atlanta, GA), a commercially available hBest2 antibody obtained from NOVUS Biologicals (Littletown, CO), anti-hBest1 mouse monoclonal (E6–6) [5], affinity-purified rabbit polyclonal anti-Best1 antibodies (pAb-125) [5] generated in our laboratory as previously described [5,6], and a monoclonal antibody (9A5) recognizing the α1 subunit of Na^+^/K^+^-ATPase (Sigma, St. Louis, MO).

### Immunoprecipitation

Immunoprecipitation of hBest2 was performed as before [9,23] using antibody GA3512 covalently coupled to Protein A beads, as described by Harlow and Lane [24]. In each experiment, ciliary bodies or RPE cells collected from four human donor eyes were lysed in 10 ml of 1% Triton X-100 in 50 mM Tris, pH 8.0, 150 mM NaCl, 1 mM EDTA, 0.2% BSA, 100 mM phenylmethylsulfonylfluoride, protease inhibitor cocktail III (1:100; EMD Chemicals, Gibbstown, NJ). Lysates were precleared with 200 μl of Pansorbin A (Calbiochem) for 1 h at 4 °C and centrifuged at 10,000× g for 5 min. The supernatant was split into 2×5 ml samples. One was incubated with 20 μl of GA3512 coupled to sephrose 4B protein A beads (ZYMED, San Francisco, CA). The other sample, a control, was incubated with uncoupled sephrose 4B protein A beads. After 1 h at 4 °C the beads were recovered by centrifugation at 5,000× g, and washed 5× in lysis buffer and 1× in 50 mM Tris, pH 8.0. Beads were then resuspended in 50 μl of Laemmli sample buffer, incubated for 5 min at 95 °C, and resolved on 10% SDS–PAGE gels. Following transfer to polyvinylidene fluoride (PVDF) membranes, Bestrophins were identified by western blotting as described previously [5]. For controls, lysates of HEK293 cells or transfected HEK293 cells expressing hBest2 were immunoprecipitated simultaneously.

### Immunofluorescence

Human donor eyes were obtained from the Lions Eyebank of Oregon. Postmortem collection times were under 6 h. Anterior segments were dissected from each globe and fixed in 4% paraformaldehyde in phosphate-buffered saline. Following cryoprotection of the tissue in 30% sucrose in phosphate-buffered saline, the tissue was embedded in Tissue Tek Optimal cutting temperature (VWR, Batavia, IL) and 10 μm sections were cut using a cryostat. HEK293 cells grown on coverslips and transfected with pAdlox-hBest2 or cryosections of human anterior segments were stained using the GA3512 antibody as described previously [25] with goat anti-rabbit Alexa Fluor 568 (Invitrogen, Eugene, OR) as a secondary antibody. In some experiments, human anterior segments were also stained with monoclonal antibody 9A5, recognizing the α1 subunit of Na^+^/K^+^ ATPase. Nuclei were labeled with 4,6'-diamidino-2-phenylindole (DAPI; Sigma).

## Results

**Figure 1:**
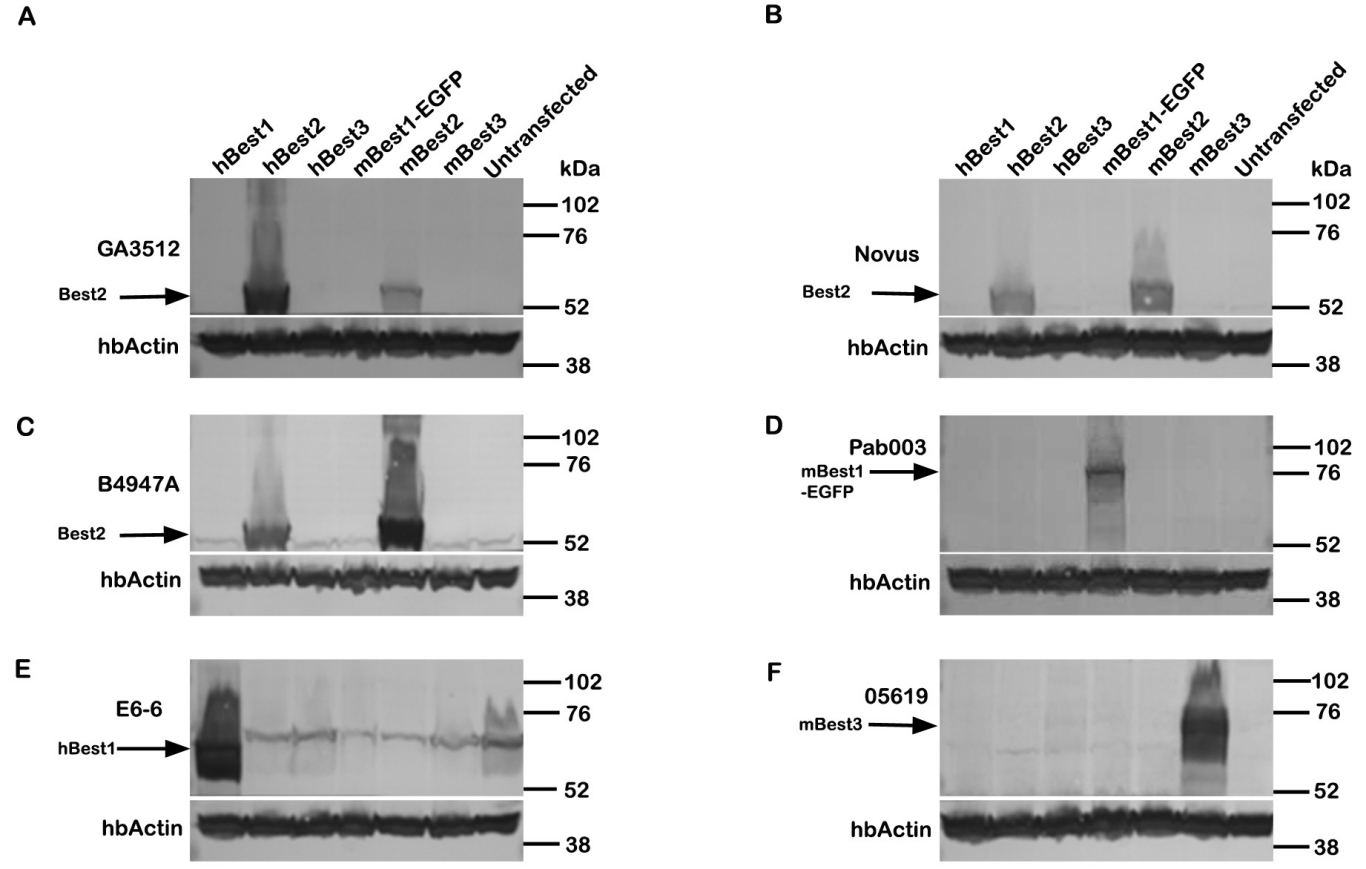
Specificity of GA3512. Western blotting was performed with cell lysates from HEK293 cells transfected to express hBest1, hBest2, hBest3, mBest1-EGFP, mBest2, or mBest3. Untransfected HEK-293 cells were included as a negative control. **A**: GA3512 specifically recognized hBest2 and mBest2, but was more efficient at identifying hBest2. It did not crossreact with other bestrophins. **B**: A commercially available anti-Best2 antibody obtained from Novus Biologicals (NOVUS) was also specific to hbest2 and mBest2. **C**: B4947A, a rabbit polyclonal antibody against mBest2 strongly recognized mBest2 and was less effective at identifying hBest2. Controls confirm expression of **D**: hBest1, **E**: mBest1-EGFP, and **F**: mBest3 using the antibodies indicated. Interestingly, only anti-Best2 antibodies recognized both the human and mouse forms. Also, note the approximately 70 kDa band that is present in every lane in **E**. This band is non-specific [1]. To insure equivalent loading, blots were cut and the bottom portion blotted for human β-actin (hb-actin).

As shown in Figure 1A, affinity-purified GA3512 recognizes recombinant hBest2 in lysates from transfected HEK293 cells, as shown by western blot. It also recognizes mBest2, though not with the same efficacy. To control for the possibility of cross-reaction with other bestrophins, we included recombinant hBest1, mBest1-EGFP, and mBest3 on our blots as well. Expression of these other bestrophins was confirmed by western blot (Figure 1D-F). GA3512, does not crossreact with human or mouse Best1 or Best3 (Figure 1A). We compared the specificity of GA3512 with a commercial rabbit anti-hBest2 polyclonal antibody (NOVUS, Biologicals, Littletown, CO; Figure 1B). The Novus antibody also was specific to Best2, but like B4947A (Figure 1C), which was raised against mBest2, it was more efficient at identifying mBest2 than hBest2.

**Figure 2:**
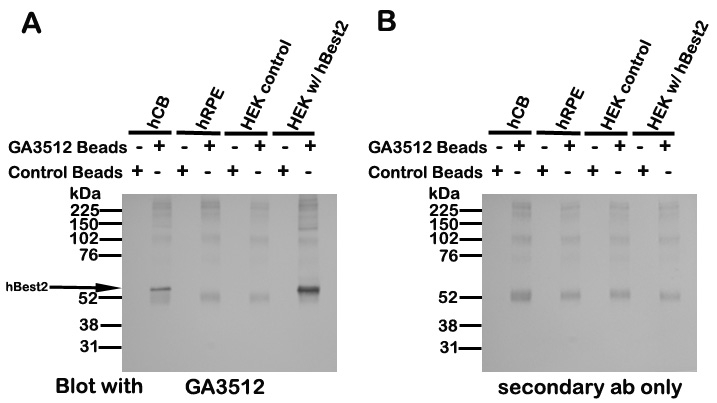
Immunoprecipitation of hBest2 from human donor tissues. **A:** Lysates of human ciliary bodies (hCBs), human RPE (hRPE) cells, HEK-293 cells (HEK control), or HEK-293 cells transfected with padlox-hBest2 (HEK w/hBest2) were immunoprecipitated with GA3512 coupled to protein A beads or control beads and blotted back with GA3512. hBest2 was identified in hCB and HEK w/hBest2 when immunoprecipitated with GA3512 beads, but not control beads. **B:** Extraneous bands in A were also present in blots when primary antibody was omitted, and likely represent IgG that has leached from the GA3512-coupled beads.

Having demonstrated the specificity of GA3512, we next tried to identify hBest2 in human ocular tissues. However, we could not identify the protein by western blot using human tissue lysates (not shown). To determine whether this was because of low levels of expression, we immunoprecipitated hBest2 using GA3512 covalently coupled to Protein A. As shown in Figure 2A, GA3512 specifically detected hBest2 in ciliary bodies and in transfected HEK293 cells, but not in human RPE cell lysates or untransfected HEK293 cells. The extraneous bands present in the immunoprecipitates were recognized by the secondary antibody used in western blotting (Figure 2B) and presumably represent IgG leaching from the GA3512-coupled beads. We conclude that hBest2 is expressed in human ciliary bodies but not in the human RPE.

**Figure 3:**
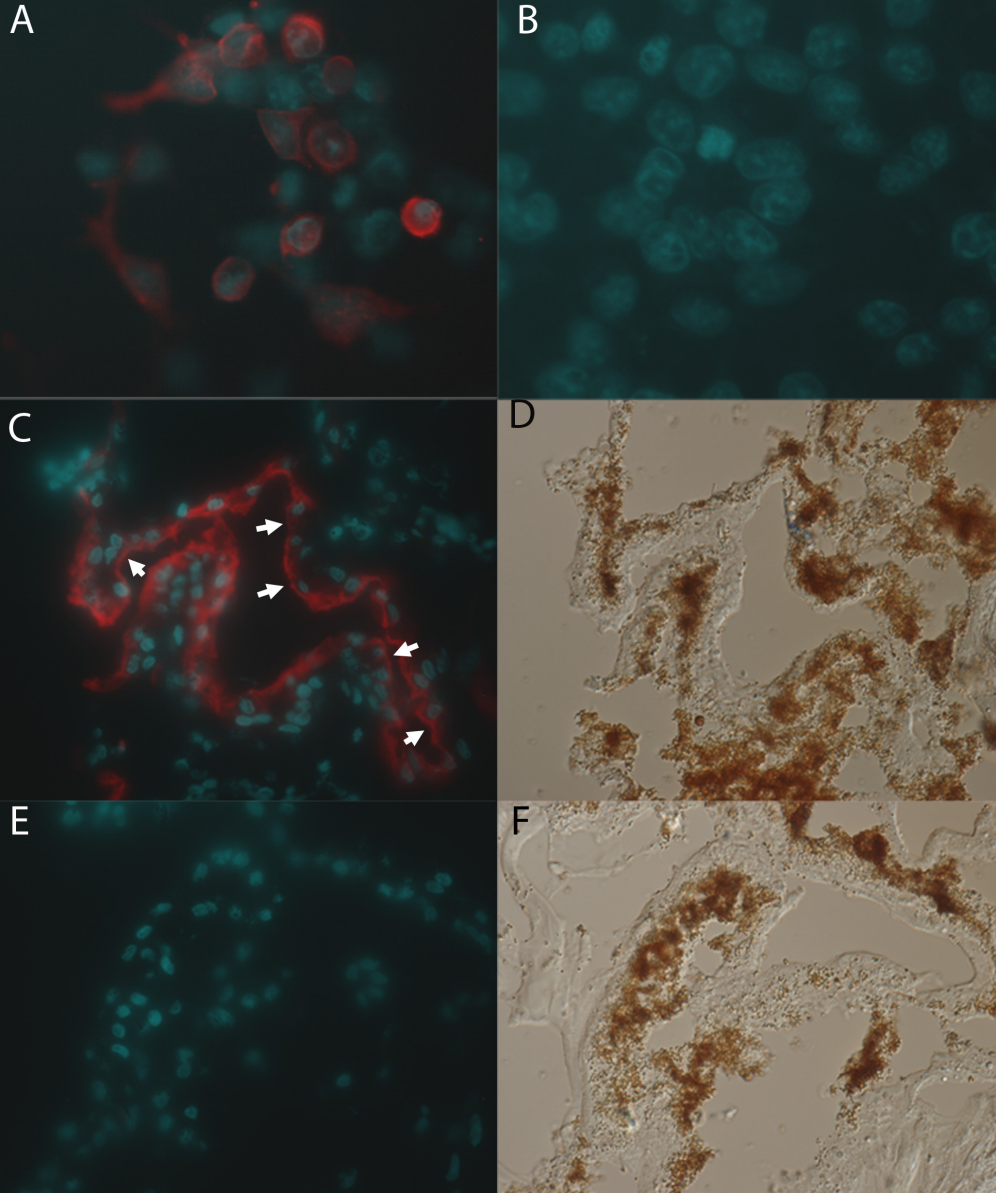
Localization of hBest2 in transfected HEK-293 cells and human ciliary body. **A**: HEK-293 cells were transfected with pAdlox-hBest2 and hBest2 localized using immunofluorescence staining with GA3512 (Red in **A, C, E**). **B**: No hBest2 staining was observed in untransfected HEK-293 cells. **C**: Although orientation varied, staining of human ciliary body identified the basolateral plasma membrane of NPE cells. Cells in which basolateral membrane staining is particularly evident are labeled by white arrows. **E**: Staining was absent when the GA3512 was omitted. **D** and **F**: are DIC micrographs of the same fields shown in **C** and **E** respectively.

We next used GA3512 to determine the localization of hBest2 in human ciliary bodies. As a precursor to these experiments, we performed immunolocalization of hBest2 in transfected HEK293 cells. GA3215 specifically detected hBest2 in the plasma membrane of transfected HEK293 cells (Figure 3A), and did not label untransfected HEK293 cells (Figure 3B). To localize hBest2 in human ciliary bodies, we stained cryosections of human anterior segments with GA3512. Using DIC optics to identify various tissues in the eye (Figure 3D), we determined that specific labeling for hBest2 was confined to NPE cells in human ciliary bodies (Figure 3C). Although the orientation of the cells varied in several cells, there was a clear separation of hBest2 staining and the DAPI-stained nuclei, suggesting its localization to the basolateral plasma membrane of the NPE. No staining was observed in control sections in which the GA3512 antibody was omitted (Figure 3E).

To more precisely localize Best2, we stained human anterior segments with GA3512 and monoclonal antibody 9A5, which recognizes the α1 subunit of Na^+^/K^+^ATPase, a marker for human ciliary epithelial plasma membranes. Inspection of the tissue using a 100× oil immersion objective (na=1.3) revealed that Best2 is predominantly located in the basal membrane of the NPE. In contrast, and as previously reported [26,27], Na^+^/K^+^ATPase stained the plasma membranes of both the pigment epithelium and NPE cells. Co-localization of Best2 and Na^+^/K^+^ATPase occurred at the basal membrane of the NPE, but nowhere else.

## Discussion

In this study, we sought to determine the location of hBest2 in the human eye. To accomplish this, we generated a novel rabbit anti-hBest2 IgG (GA3512) that is specific to Best2 (Figure 1) and preferentially recognizes hBest2 over mBest2. GA3512 demonstrated a high specificity and titer in western blotting, immunolocalization, and immunoprecipitation assays.

**Figure 4:**
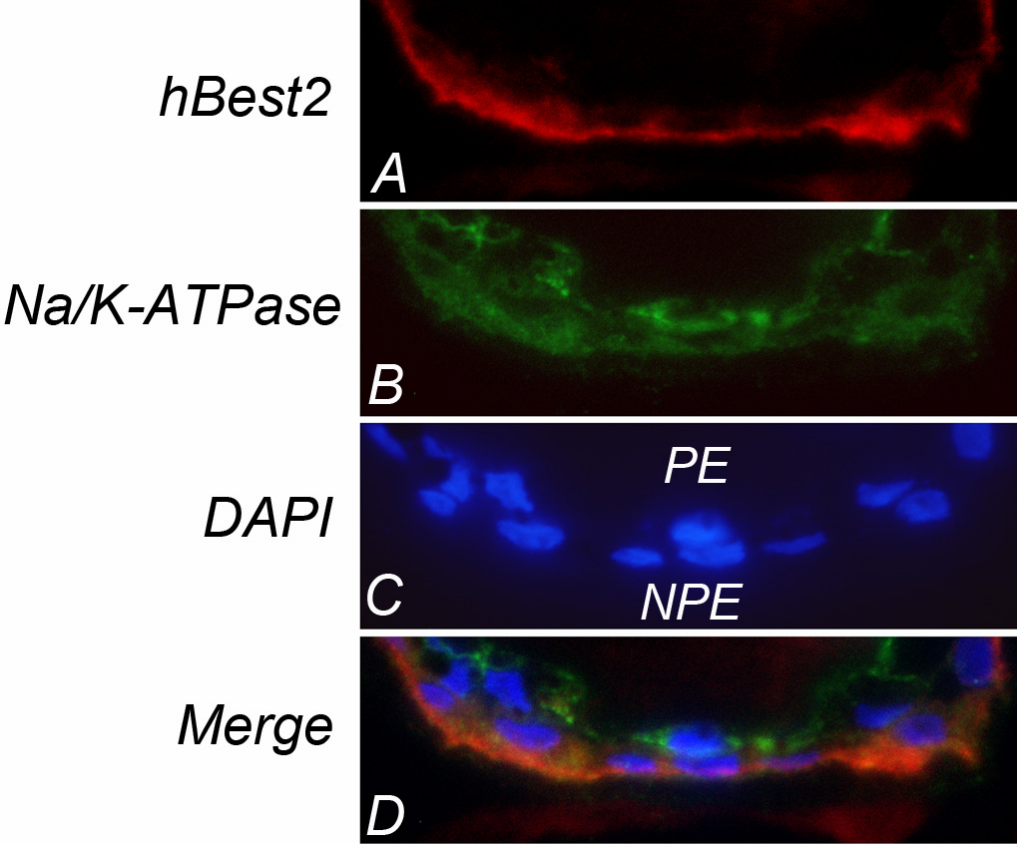
Co-localization of hBest2 with the α1 subunit of Na^+^/K^+^-ATPase at the basal plasma membrane of the RPE. **A:** Cryosections of human anterior segments were reacted with GA3512 to identify hBest2. **B:** The localization of Na^+^/K^+^-ATPase was determined using a monoclonal antibody against the α1 subunit. **C:** Nuclei were stained with DAPI. **D:** A merge of the images shows that hBest2 co-localizes with Na^+^/K^+^-ATPase at the basal surface of the NPE. note that the α1 subunit of Na^+^/K^+^-ATPase is also found in the plasma membrane of PE cells, where hBest2 is not expressed.

Having validated the specificity of GA3512, we tried to determine whether hBest2 is expressed in the NPE, as mBest2 is in the mouse. We have previously shown by RT–PCR that hBest2 mRNA is found in human ciliary bodies [4]. Unfortunately, western blotting proved unsuccessful at identifying hBest2 in any ocular tissue. We hypothesized that this was due to a low level of hBest2 protein expression, and attempted to enrich hBest2 by immunoprecipitation. This proved successful, and we were able to identify hBest2 in immunoprecipitates from human ciliary bodies. We did not observe hBest2 in immunoprecipitates from human RPE cells (Figure 2). We next used GA3512 to determine the specific location of hBest2. As shown previously for mBest2 [4], in transfected HEK293 cells, hBest2 is localized within the plasma membrane (Figure 3A). Immunofluorescence staining of human donor eyes identified hBest2 staining only in the ciliary bodies (Figure 3C), where it appeared to be uniquely expressed in NPE cells. We sought to more precisely locate hBest2 in the NPE by comparing its localization in the α1 subunit of Na^+^/K^+^-ATPase (Figure 4). Similar to previous reports [26,27], we found that in human ciliary bodies, the α1 subunit of Na^+^/K^+^-ATPase is localized in the basolateral plasma membrane of both PE and NPE cells (Figure 4B,D). In contrast, hBest2 staining co-localized with Na^+^/K^+^-ATPase only at the basolateral plasma membrane of NPE cells (Figure 3 and Figure 4A,D). This is the same location that we have previously shown for mBest2 in mice [4].

The phenotype of *Best2^−/−^* mice is a diminished IOP [4,18]. Determination of aqueous dynamics in *Best2^−/−^* mice indicates that aqueous flow, however, is increased. The diminished IOP results from overcompensation of the outflow pathways [18], indicating that in the mouse, Best2 plays a role in regulating IOP by affecting both inflow and outflow. The findings reported herein, that hBest2, like mBest2, is located in the basal plasma membrane of NPE cells in humans, suggests that Best2 may also be an important player in regulating IOP in humans. Consequently, these data support the hypothesis that modulation of Best2 activity may represent a new therapeutic avenue for lowering IOP in individuals with glaucoma.
